# High Level of Uric Acid Promotes Atherosclerosis by Targeting NRF2-Mediated Autophagy Dysfunction and Ferroptosis

**DOI:** 10.1155/2022/9304383

**Published:** 2022-04-18

**Authors:** Wei Yu, Weidong Liu, De Xie, Qiang Wang, Chenxi Xu, Hairong Zhao, Jiaming Lv, Furong He, Bingyang Chen, Tetsuya Yamamoto, Hidenori Koyama, Jidong Cheng

**Affiliations:** ^1^Department of Internal Medicine, Xiang'an Hospital of Xiamen University, School of Medicine, Xiamen University, Xiamen, Fujian, China; ^2^Department of Diabetes, Endocrinology and Clinical Immunology, Hyogo College of Medicine, Nishinomiya, Hyogo, Japan; ^3^Xiamen Key Laboratory of Translational Medicine for Nucleic Acid Metabolism and Regulation, Xiamen, Fujian, China

## Abstract

Atherosclerotic vascular disease (ASVD) is the leading cause of death worldwide. Hyperuricemia is the fourth risk factor for atherosclerosis after hypertension, diabetes, and hyperlipidemia. The mechanism of hyperuricemia affecting the occurrence and development of atherosclerosis has not been fully elucidated. Mononuclear macrophages play critical roles in all stages of atherosclerosis. Studies have confirmed that both hyperuricemia and ferroptosis promote atherosclerosis, but whether high level of uric acid (HUA) promotes atherosclerosis by regulating ferroptosis in macrophages remains unclear. We found that HUA significantly promoted the development of atherosclerotic plaque and downregulated the protein level of the NRF2/SLC7A11/GPX4 signaling pathway in ApoE^−/−^ mice. Next, we evaluated the effect of HUA and ferroptosis inhibitor ferrostatin-1 (Fer-1) treatment on the formation of macrophage-derived foam cells. HUA promoted the formation of foam cells, decreased cell viability, and increased iron accumulation and lipid peroxidation in macrophages treated with oxidized low-density lipoprotein (oxLDL); these effects were reversed by Fer-1 treatment. Mechanistically, HUA significantly inhibited autophagy and the protein level of the NRF2/SLC7A11/GPX4 signaling pathway. Fer-1 activated autophagy and upregulated the level of ferroptosis-associated proteins. Moreover, an NRF2 inducer (tertbutyl hydroquinone (TBHQ)) and autophagy activator (rapamycin (RAPA)) could reverse the inhibitory effect of HUA on foam cell survival. Our results suggest that HUA-induced ferroptosis of macrophages is involved in the formation of atherosclerotic plaques. More importantly, enhancing autophagy and inhibiting ferroptosis by activating NRF2 may alleviate HUA-induced atherosclerosis. These findings might contribute to a deeper understanding of the role of HUA in the pathogenesis of atherosclerosis and provide a therapeutic target for ASVD associated with hyperuricemia.

## 1. Introduction

Hyperuricemia is a metabolic disorder syndrome caused by purine nucleotide metabolism disorder. The overall prevalence of hyperuricemia in China is 13.3%, and it has become a common metabolic disease after diabetes [1, 2]. Hyperuricemia is not only an independent risk factor for cardiovascular disease but also an independent predictor of all-cause mortality [3–6]. For each 1 mg/dl increase in serum uric acid (sUA) level, the risk of death from atherosclerotic vascular disease (ASVD) is increased by 48% in men and 126% in women [7]. However, the molecular mechanisms of high level of uric acid (HUA) affecting the occurrence and development of ASVD are far from being clarified.

Ferroptosis is a newly discovered nonapoptotic-regulated cell death characterized by iron accumulation and lipid peroxidation [8–10]. The ferroptosis of macrophage-derived foam cells plays a crucial role in the formation and development of atherosclerotic plaques [11–14]. Glutathione peroxidase 4 (GPX4) is considered one of the most important antioxidant enzymes because of its unique ability to reduce phospholipid hydroperoxide activity [15, 16]. Inactivation of GPX4 in mice and cells led to excessive lipid peroxidation, thus triggering ferroptotic cell death [14, 15, 17]. In contrast, GPX4 overexpression removed oxidative lipid modifications and inhibited plaque formation in ApoE^−/−^ mice [18]. Ferrostatin-1 (Fer-1), a selective inhibitor of erastin-induced ferroptosis, could inhibit atherosclerosis progression *in vivo* [13]. However, whether HUA can promote the occurrence and development of atherosclerosis by regulating ferroptosis in macrophage-derived foam cells remains unclear.

Nuclear factor erythroid 2-related factor 2 (NFE2L2/NRF2) is a key regulator of antioxidant responses because many of its downstream target genes are involved in preventing or correcting redox imbalance in cells [19, 20]. NRF2 also plays an essential role in many key metabolic pathways, including iron/heme metabolism, proteostasis, carbohydrate and lipid metabolism, and apoptosis [21–26]. Thus, NRF2 has emerged as a major regulator of lipid peroxidation and ferroptosis. GPX4 and the cysteine/glutamate transporter system xC^−^/xCT/SLC7A11 are important downstream regulatory targets of NRF2 [9, 26, 27]. Recent studies have indicated that NRF2 is involved in regulating some genes related to macrophage autophagy, such as p62 and Atg5 [28–32]. Treating ApoE^−/−^ mice with tertbutyl hydroquinone (TBHQ), an NRF2 inducer, had a protective effect on atherosclerosis [33]. The molecular mechanisms are by upregulating antioxidant and anti-inflammatory effects and by enhancing the autophagic flux of macrophages in plaque [33]. Therefore, ferroptosis is considered an autophagic cell death process, with NRF2-mediated autophagy playing a crucial role [34, 35]. Recent study confirmed that HUA inhibits NRF2 signaling and promotes the production of excessive reactive oxygen species (ROS) in chicken embryo cardiomyocytes [36]. Therefore, we speculated that NRF2-mediated ferroptosis may contribute to the occurrence and development of hyperuricemia-associated ASVD.

In this study, we determined the effects and underlying mechanisms of HUA on the transition of macrophage phenotypes and the formation of atherosclerosis in ApoE^−/−^ mice and macrophages treated with oxidized low-density lipoprotein (oxLDL). HUA impaired mitochondrial function, inhibited NRF2 signaling and autophagy, increased ferroptosis, and thus promoted the formation of atherosclerotic plaques; all effects were reversed by treatment with Fer-1.

## 2. Materials and Methods

### 2.1. Antibodies and Chemicals

Rabbit anti-NRF2 (Cat# ab62352), Alexa Fluor 488 anti-rabbit secondary antibody (Cat# ab150077), and iron assay kit (Cat# ab83366) were from Abcam (UK). Anti-LC3B (Cat# 2775) and anti-p62 (Cat# 39749) antibodies were from CST (USA). GPX4 (Cat# A1933), SLC7A11 (Cat# A15604), CD68 (Cat# A13286), GAPDH (Cat# AC002), and *β*-actin (Cat# AC026) antibodies and C11-BODIPY (Cat# RM02821) were from ABclonal (Wuhan, China). Phorbol 12-myristate 13-acetate (PMA) (Cat# 16561-29-8), UA (Cat# 69-93-2), and RAPA (Cat# 53123-88-9) were from Sigma (USA). Fer-1 (Cat# HY-100579) and TBHQ (Cat# HY-100489) were from MCE (USA). Human oxLDL (Cat# YB-002) was from Yiyuan Biotechnologies (Guangzhou, China).

### 2.2. Animals and Treatment

ApoE^−/−^ mice (C57BL/6J background) were purchased and fed in the Laboratory Animal Center of Xiamen University (Fujian, China). Eight-week-old male ApoE^−/−^ mice were randomly divided into three groups: standard laboratory diet (SLD) group (fed a standard laboratory diet for 16 weeks, *n* = 6), high fat diet (HFD) group (fed a fat-rich Western diet (provided 37% kcal in fat, Cat# HD012a, Botai, Beijing) for 16 weeks, *n* = 6), and HUA and high fat diet (HUA+HFD) group (fed a fat-rich Western diet for 16 weeks and intraperitoneally injected with hypoxanthine (100 mg/kg) and oteracil potassium (150 mg/kg) once every two days for the last 3 weeks, *n* = 6).

All experimental procedures and animal housing in this study were designed and conducted in accordance with the approval of the Institutional Animal Care and Use Committee of Xiamen University, China (Animal Ethics No. XMULAC20200122).

### 2.3. Serum Biochemical Profile Detection

Biochemical kits were used to detect sUA (Cat# C102) and lipid profiles, including total cholesterol (TC) (Cat# A111), triglycerides (TG) (Cat# A110), and high-density lipoprotein-cholesterol (HDL-C) and low-density lipoprotein-cholesterol (LDL-C) (Cat# A112 and A113, respectively), following the manufacturer's instructions. The supernatant of the mice blood samples was centrifuged and determined by using a microplate reader (Thermo, USA). All Biochemical kits were from Nanjing Jiancheng Bioengineering Institute (Nanjing, China).

### 2.4. Cell Cultures and Treatment

THP-1 cells and RAW264.7 cells were obtained from the American Type Cell Collection and grown in RPMI-1640 and DMEM medium (Gibco, Shanghai) containing 10% fetal bovine serum (Gibco, Shanghai). RAW264.7 cells were incubated with DMEM, and THP-1 cells were primed with 160 nM PMA for 24 h, then exposed to oxLDL (100 *μ*g/ml), or coincubated with UA (15 mg/dl) and oxLDL for another 24 h. In experiments involving inhibitors or activators, the cells were pretreated with Fer-1 (2 *μ*M), TBHQ (10 *μ*M), or RAPA (10 *μ*M) for 0.5 h, and then, oxLDL and/or UA was added for another 24 h.

### 2.5. Oil Red O Staining

To observe atherosclerotic plaque formation *in vivo* and the fat accumulation *in vitro*, the cells were exposed to various treatments, and frozen serial cross-sections of aortic tissue were stained with Oil red O (Cat# C0158S, Beyotime, China) [37]. ImageJ was used to analyze atherosclerotic lesion area in 3 cross-sections/mouse (*n* = 5).

### 2.6. Immunohistochemical Staining

To assess the infiltration of macrophages in the atherosclerotic lesion area of the aortic sinus, 3 cross-sections/mouse (*n* = 3) underwent immunohistochemical staining with anti-CD68 antibody (1 : 100 dilution) [37]. ImageJ software was used for data analyses.

### 2.7. Immunofluorescence Microscopy

Frozen sections of aortic tissue (3 cross-sections/mouse, *n* = 3) were fixed, permeabilized, and blocked, and the primary antibodies (NRF2, SLC7A11, and GPX4, 1 : 100 dilution) were added for incubation overnight at 4°C. After a wash the next day, the cells were incubated with Alexa Fluor 488 anti-rabbit secondary antibody (1 : 1000 dilution) at room temperature and out of light for 1 h and then incubated with DAPI (4′,6′-diamidino-2-phenylindole) in 1% goat serum for 5 min at room temperature. The cells were imaged by laser scanning confocal microscopy (FV1000 MPE-B, Olympus, Tokyo, Japan). ImageJ software was used for data analyses.

### 2.8. Cell Viability Assay

Cell viability was evaluated by using the cell counting kit-8 (CCK-8) (Cat# K1018, APExBIO, USA) according to the manufacturer's instructions. Briefly, the cells were seeded into 96-well plates at a density of 5 × 103 cells per well and incubated for 24 h before being subjected to various treatments. Following that, 10 *μ*l of CCK8 solution was added to each well and cultivated for 2 hours. The absorbance at 450 nm was measured by a microplate reader (Multiskan Skyhigh, Thermo, USA).

### 2.9. Malondialdehyde (MDA) Assay

Relative MDA concentration in cell lysates was assessed with a lipid peroxidation assay kit (Cat# A003-1) purchased from Nanjing Jiancheng Bioengineering Institute (China) according to the manufacturer's instructions. Briefly, after adding TBA to cell lysates, the absorbance was recorded at 532 nm using a microplate reader to calculate the MDA level.

### 2.10. Iron Assay

Intracellular ferrous iron (Fe^2+^) level was determined by using an iron assay kit (Abcam, Cat# ab83366) according to the manufacturer's instructions. The cells were lysed on ice, and supernatants were collected and coincubated with 5 *μ*l Iron Reducer solution at 37°C for 30 min. Then, 100 *μ*l Iron Probe was added and incubated in darkness at 37°C for 1 h. The absorbance was measured at 593 nm with a microplate reader.

### 2.11. Lipid ROS Assay

Lipid ROS level was detected by flow cytometry (FCM) with BODIPY-C11 dye. In brief, the cells were treated as indicated; then, 50 *μ*M C11-BODIPY (ABclonal, Cat# RM02821) was added and incubated for 1 h. Lipid ROS generation was analyzed by FCM according to the manufacturer's instructions.

### 2.12. Glutathione (GSH) Assay

The relative GSH level in cell lysates was analyzed by using a GSH assay kit (Cat# A006-2, Nanjing Jiancheng Bioengineering Institute) according to the manufacturer's instructions. RAW264.7 and THP-1 cells were seeded at 5 × 105 cells in 6-well plates. After 24 h of treatment with different methods, the supernatant of cell lysates was taken and GSH level was determined according to the method provided by the assay kit. The absorbance was measured at 410 nm on a microplate reader.

### 2.13. Transmission Electron Microscopy (TEM)

After treatment, cell specimens were fixed with 2.5% glutaraldehyde and sliced into ultrathin slices. The slices were then stained with uranyl acetate and lead citrate. Finally, put the slice under the TEM (HT-7800; Hitachi, Tokyo) and investigate the cell ultrastructure.

### 2.14. Measurement of Mitochondrial Membrane Potential (MMP)

MMP was measured by using the JC-1 staining assay kit (Cat# C2006, Beyotime, China) according to the manufacturer's instructions. Briefly, the cells were incubated with JC-1 fluorescent probe for 20 minutes in the dark at 37°C. After washing with PBS, cell images were detected using a fluorescent microscope (IX83, Olympus Co., Japan). The average fluorescence intensity (AFI) was quantified by using ImageJ.

### 2.15. RNA Extraction, cDNA Synthesis, and qPCR Analysis

Total RNA was isolated with the RNeasy Mini Kit (Cat# B511311, Sangon Biotech, Shanghai), and 2 *μ*g of total RNA was used for cDNA synthesis with the PrimeScript RT-PCR Kit (Cat# KR106, TIANGEN, Beijing). Quantitative PCR involved using the Hieff qPCR SYBR Green Master Mix (Cat# 11201, Yeason, Shanghai). Samples were obtained and analyzed on the CFX96 Touch Quantitative PCR (qPCR) Detection System (Bio-Rad, USA). Gene levels were normalized to *β*-actin level. The primer sequences are in Table 1.

### 2.16. Immunoblotting

The cells were trypsinized and washed twice with ice-cold PBS and then lysed in radioimmunoprecipitation lysis buffer (Cat# EA0002, Sparkjade Biotechnology, China) with protease and phosphatase inhibitors. Cell lysates were resolved by 13% SDS-PAGE and transferred to polyvinylidene difluoride (PVDF) membranes (Millipore, Billerica, MA, USA), which were incubated with the indicated primary antibodies (1 : 1000 dilution) at 4°C overnight and then HRP-conjugated secondary antibodies (1 : 2000 dilution) for 1 h at room temperature. The protein bands were imaged by using the Enhanced Chemiluminescence Kit (Cat# 34580, Thermo, USA), and detection involved using Azure Biosystems C300 (USA). The band intensities were quantified by using ImageJ.

### 2.17. Statistical Analysis

Data are presented as means ± SD. Unpaired Student's *t* test was used to compare two groups and one-way ANOVA to compare multiple groups. All data were analyzed by using GraphPad Prism 8.0 (GraphPad Software, Inc.). *P* < 0.05 was considered statistically significant.

## 3. Results

### 3.1. HUA Promotes the Development of Atherosclerosis in ApoE^−/−^ Mice

**Figure 1:**
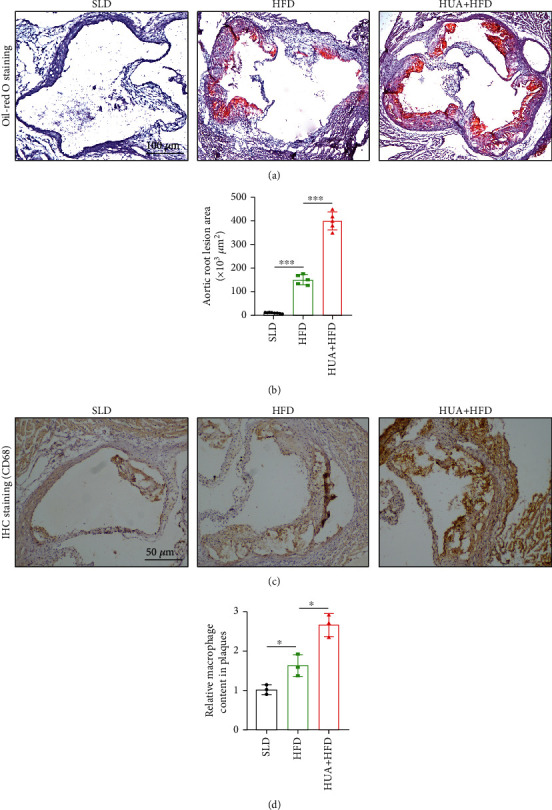
HUA promotes atherosclerosis development in ApoE^−/−^ mice. Eight-week-old male ApoE^−/−^ mice were randomly divided into three groups (SLD, HFD, and HUA+HFD); when the mice reached age 22 weeks (after 14 weeks with various treatment), aortas were dissected. (a) Oil red O staining of frozen aortic root sections from the 3 groups. (b) Quantification of the aortic root lesion areas (×10^3^*μ*m^2^). Data are means ± SD, 3 cross-sections/mouse, *n* = 5. (c) Macrophage infiltration in the aortic root sections examined by IHC staining with an anti-CD68 antibody and (d) quantification. Data are means ± SD, 3 cross-sections/mouse, *n* = 3. HFD: high fat diet; HUA: high level of uric acid; HUA+HFD: high level of uric acid and high fat diet group; IHC: immunohistochemical; SLD: standard laboratory diet. ^∗^*P* < 0.05 and ^∗∗∗^*P* < 0.001.

To explore the pathophysiological roles of HUA in atherosclerosis, ApoE^−/−^ mice were given SLD, HFD, or HUA+HFD treatment. sUA levels, body weight, serum lipid profile, and atherosclerotic lesion formation were assessed after 16 weeks of various treatment. As compared with HFD mice, HUA+HFD mice showed slightly decreased body weight, but a 2.17-fold increase in sUA level (Table 2). HUA+HFD mice showed elevated HLD-C level, with decreased LDL-C level (Table 2). Oil red O staining of the aortic sinus showed a significant increase in lipid accumulation in HUA+HFD versus HFD mice (Figure 1(a)). Plaque lesion area in the aortic sinus was 2.67-fold greater in HUA+HFD than HFD mice (Figure 1(b)). These results suggest that HUA may play a critical role in the aggravation of atherosclerosis. Likewise, immunohistochemical results showed that macrophage infiltration in the atherosclerotic lesion area of the aortic sinus was significantly higher in HUA+HFD than HFD mice (Figures 1(c) and 1(d)).

### 3.2. HUA Inhibits the Protein Level of the NRF2/SLC7A11/GPX4 Signaling Pathway in Macrophages in Atherosclerotic Plaques

**Figure 2:**
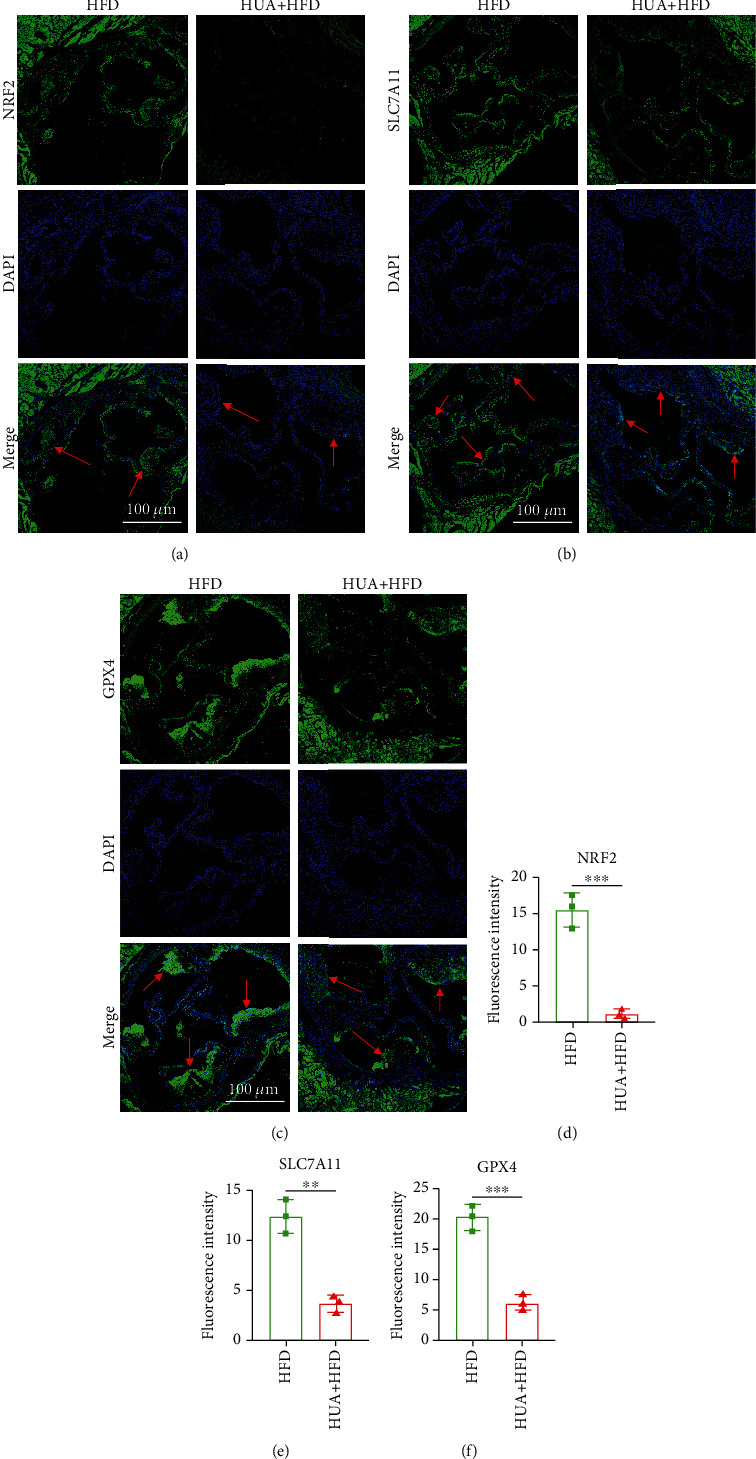
HUA inhibits the protein level of the NRF2/SLC7A11/GPX4 signaling pathway in atherosclerotic plaque from HFD-treated ApoE^−/−^ mice. The protein level of the NRF2/SLC7A11/GPX4 signaling pathway was assessed by immunofluorescence staining. (a–c) Representative immunostaining of NRF2, SLC7A11, and GPX4 in aortic atherosclerotic lesions in HUA+HFD versus HFD group. (d–f) Quantification of the mean fluorescence intensity for NRF2, SLC7A11, and GPX4. Data are means ± SD, 3 cross-sections/mouse, *n* = 3. HFD: high fat diet; HUA: high level of uric acid; HUA+HFD: high level of uric acid and high fat diet group. ^∗∗^*P* < 0.01 and ^∗∗∗^*P* < 0.001.

Ferroptosis of macrophage-derived foam cells plays a crucial role in the formation and development of atherosclerotic plaques [11, 12, 38–41]. The NRF2/SLC7A11/GPX4 signaling pathway is one of the most important defense systems for ferroptosis [21, 23, 26, 27, 42–45]. To this end, we used immunofluorescence staining to detect the protein level of the NRF2/SLC7A11/GPX4 signaling pathway in aortic plaque. HUA significantly inhibited the protein level of the signaling pathway (Figures 2(a)–2(f)), which suggests that NRF2-mediated ferroptosis is involved in the progression of hyperuricemia-associated ASVD.

### 3.3. HUA Promotes Macrophage-Derived Foam Cell Formation in THP-1 and RAW264.7 Cells

**Figure 3:**
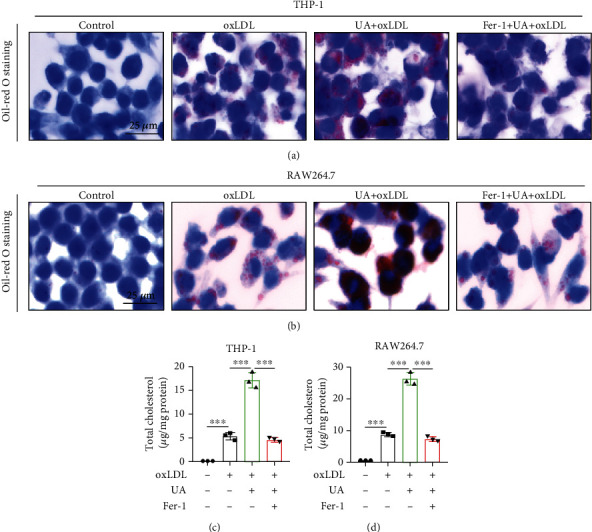
HUA promotes macrophage-derived foam cell formation in THP-1 and RAW264.7 cells. THP-1 and RAW264.7 cells were treated with oxLDL (100 *μ*g/ml) or coincubated with UA (15 mg/dl) and with or without Fer-1 (2 *μ*M) for 24 h. (a and b) Oil red O staining to evaluate the effect of HUA and Fer-1 on the formation of foam cells. (c and d) Quantification of the lipid accumulation in THP-1 and RAW264.7 cells. Data are means ± SD, *n* = 3. Fer-1: ferrostatin-1; HUA: high level of uric acid; oxLDL: oxidized low-density lipoprotein; UA: uric acid. ^∗∗∗^*P* < 0.001.

Macrophage-derived foam cell formation is a crucial step in the pathogenesis of atherosclerosis [46]. We evaluated the effect of HUA on the formation of macrophage-derived foam cells. THP-1 and RAW264.7 cells were exposed to oxLDL (100 *μ*g/ml) or coincubated with UA (15 mg/dl) and oxLDL for 24 h. Oil red O staining revealed that HUA enhanced foam cell formation (Figures 3(a) and 3(b)) and increased lipid accumulation (Figures 3(c) and 3(d)). Thus, HUA promoted foam cell formation in THP-1 and RAW264.7 cells.

### 3.4. HUA-Induced Ferroptosis Is a Key Regulatory Factor Promoting Foam Cell Formation

**Figure 4:**
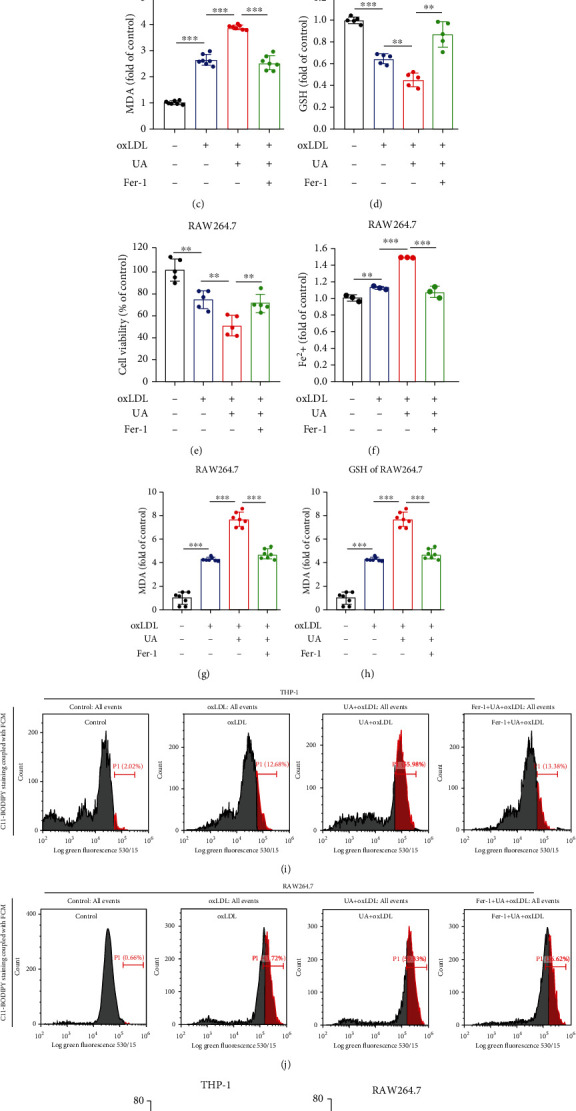
HUA-induced ferroptosis is a key regulatory promoting foam cell formation. THP-1 and RAW264.7 cells were treated with oxLDL (100 *μ*g/ml) or coincubated with UA (15 mg/dl) with or without Fer-1 (2 *μ*M) for 24 h. (a and e) Cell viability was assayed by using a CCK-8 kit. (b and f) The accumulation of Fe^2+^ was measured by an iron detection assay. (c and g) Lipid formation was measured by MDA assay. (d and h) Relative GSH level was detected by using an assay kit. (i and j) C11-BODIPY staining coupled with FCM was used to assess lipid ROS levels. (k and l) Quantification of lipid ROS levels. Data are means ± SD, *n* = 3 − 7. FCM: flow cytometry; Fer-1: ferrostatin-1; GSH: glutathione; HUA: high level of uric acid; MDA: malondialdehyde; oxLDL: oxidized low-density lipoprotein; ROS: reactive oxygen species; UA: uric acid. ^∗∗^*P* < 0.01 and ^∗∗∗^*P* < 0.001.

Ferroptosis has been associated with various tissue and organ diseases, including neurodegenerative diseases, acute kidney injury, myocardial ischemia–reperfusion injury, and ASVD [11, 26, 38, 39, 41]. To gain insights into the potential mechanisms by which HUA promotes foam cell formation, we assessed the ferroptosis of foam cells treated with UA (15 mg/dl). HUA treatment promoted oxLDL-induced cell death in THP-1 and RAW264.7 cells (Figures 4(a) and 4(e)), along with increased ferroptotic events including iron accumulation (Figures 4(b) and 4(f)), MDA production (Figures 4(c) and 4(g)), GSH depletion (Figures 4(d) and 4(h)), and lipid ROS production (Figures 4(i)–4(l)). In contrast, pretreatment with Fer-1 increased cell viability in oxLDL-treated macrophages owing to reduced ferroptotic events (Figures 4(a)–4(l)). Intriguingly, Fer-1 also reversed HUA-enhanced foam cell formation (Figures 3(a) and 3(b)) and reversed the increased lipid accumulation (Figures 3(c) and 3(d)). Therefore, HUA specifically regulates oxLDL-induced ferroptosis in macrophages and promotes foam cell formation by ferroptosis.

### 3.5. Mitochondrial Damage Contributes to HUA-Induced Ferroptosis in Foam Cells

**Figure 5:**
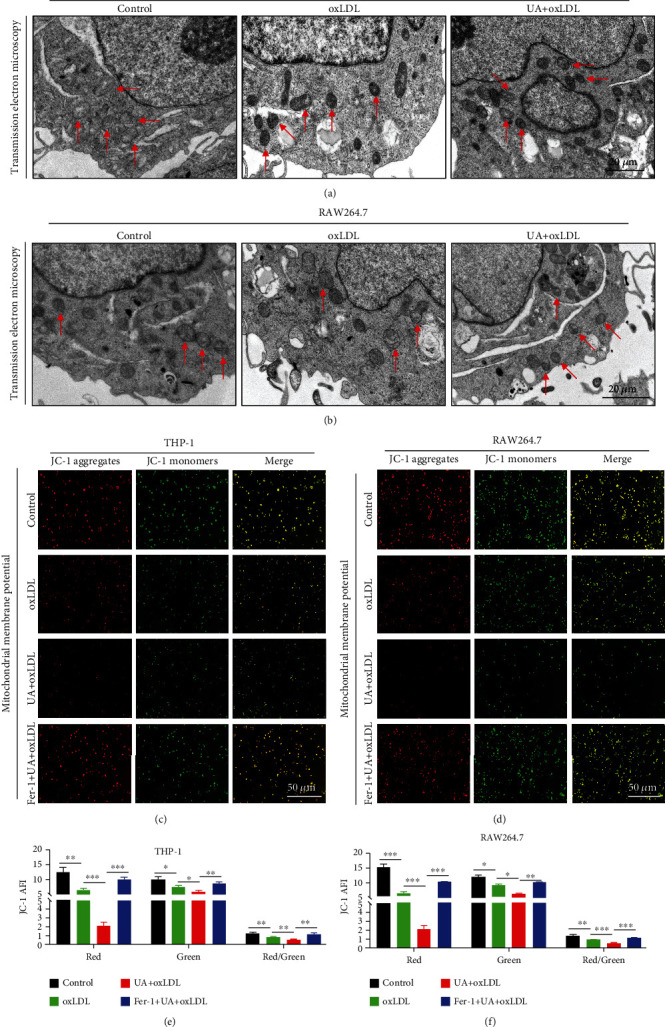
Mitochondrial damage contributes to HUA-induced ferroptosis in foam cells. THP-1 and RAW264.7 cells were treated with oxLDL (100 *μ*g/ml) or coincubated with UA (15 mg/dl) with or without Fer-1 (2 *μ*M) for 24 h. (a and b) TEM images of macrophages treated with oxLDL or coincubated with UA. Arrows indicate mitochondria. (c and d) A JC-1 staining kit was used to assess MMP level in THP-1 and RAW264.7 cells. (e and f) Quantification of MMP level. Data are means ± SD, *n* = 3. AFI: average fluorescence intensity; Fer-1: ferrostatin-1; HUA: high level of uric acid; MMP: mitochondrial membrane potential; oxLDL: oxidized low-density lipoprotein; TEM: transmission electron microscopy; UA: uric acid. ^∗^*P* < 0.05, ^∗∗^*P* < 0.01, and ^∗∗∗^*P* < 0.001.

In addition to iron overload and lipid peroxidation, mitochondrial changes are another major feature of ferroptosis. Accordingly, we observed mitochondrial ultrastructural changes in THP-1 and RAW264.7 cells treated with oxLDL or coincubated with UA for 24 h. TEM results showed that mitochondria were generally smaller and mitochondrial cristae were reduced in the UA-treated group (Figures 5(a) and 5(b)). These changes in mitochondrial structure may lead to mitochondrial dysfunction. To further confirm the effect of UA on mitochondrial function in foam cells, we examined the MMP by JC-1 staining. The red average fluorescence intensity (AFI) and red/green fluorescence ratio were significantly reduced after UA treatment (Figures 5(c)–5(f)), so the MMP was reduced in foam cells. However, green AFI was not significantly increased as expected. These phenomena were partially alleviated by Fer-1 treatment (Figures 5(c)–5(f)). These data indicate that HUA induced mitochondrial dysfunction, which contributed to inducing ferroptosis in macrophage-derived foam cells.

### 3.6. NRF2-Mediated Autophagy Dysfunction and Ferroptosis Are Involved in Foam Cell Formation Induced by HUA

**Figure 6:**
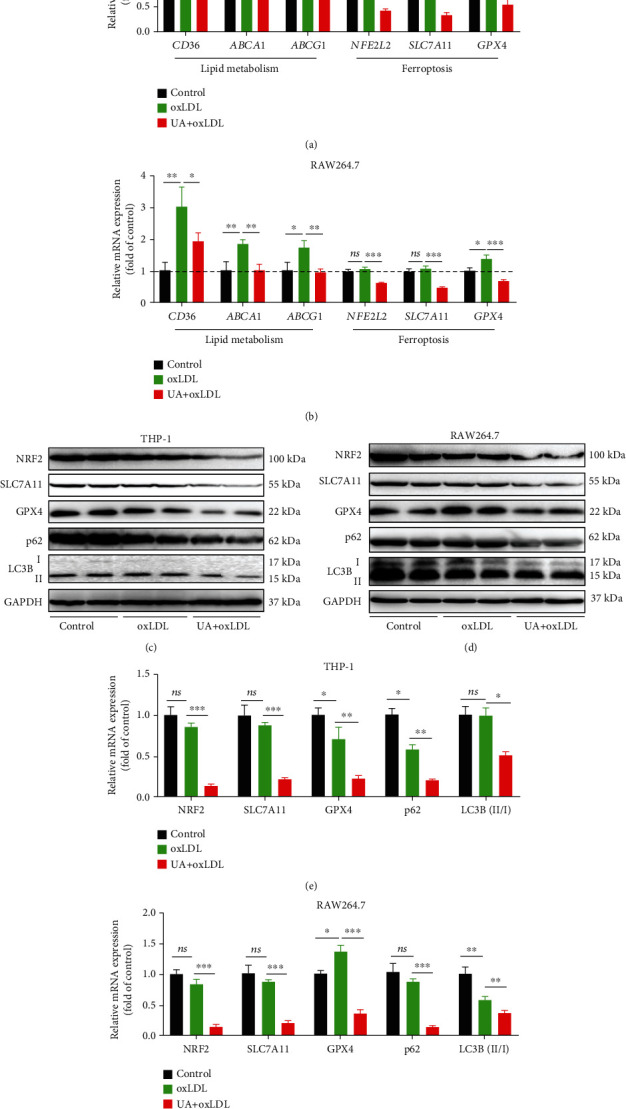
NRF2-mediated autophagy dysfunction and ferroptosis are involved in foam cell formation induced by HUA. THP-1 and RAW264.7 cells were treated with oxLDL (100 *μ*g/ml) or coincubated with UA (15 mg/dl) for 24 h. (a and b) qPCR was used to detect the transcription of lipid metabolism-related genes (*CD36*, *ABCA1*, and *ABCG1*) and ferroptosis-related genes (*NFE2L2*, *SLC7A11*, and *GPX4*) in macrophage-derived foam cells. (c and d) Representative western blot images of the protein level of the NRF2/SLC7A11/GPX4 signaling pathway and autophagy-related proteins (LC3B and p62). (e and f) Quantification of NRF2, SLC7A11, GPX4, LC3B, and p62 protein levels. Data are means ± SD, *n* = 3. ABCA1: ATP-binding cassette transporter A1; ABCG1: ATP-binding cassette transporter G1; HUA: high level of uric acid; oxLDL: oxidized low-density lipoprotein; qPCR: quantitative PCR. ns indicates no significance. ^∗^*P* < 0.05, ^∗∗^*P* < 0.01, and ^∗∗∗^*P* < 0.001.

GPX4 and SLC7A11 are central regulators of ferroptosis as well as downstream target genes of NRF2 [21, 22, 27, 44]. To investigate whether the NRF2/SLC7A11/GPX4 signaling pathway was regulated by HUA in foam cells, we detected the expression of the pathway by qPCR and western blot analysis. HUA decreased the protein level of the NRF2/SLC7A11/GPX4 signaling pathway in macrophage-derived foam cells (Figures 6(c)–6(f)), which was further confirmed by qPCR results (Figures 6(a) and 6(b)). In addition, qPCR results showed that HUA inhibited the transcription of lipid metabolism-related genes such as *CD36*, ATP-binding cassette transporter A1 (*ABCA1*), and ATP-binding cassette transporter G1 (*ABCG1*) (Figures 6(a) and 6(b)). Notably, HUA also suppressed the protein level of LC3B and p62, which are markers of autophagic status (Figures 6(c)–6(f)). Collectively, these data suggest that NRF2-mediated autophagy dysfunction and ferroptosis may be involved in foam cell formation associated with HUA.

### 3.7. Ferroptosis Inhibitor (Fer-1) Reverses HUA-Induced Foam Cell Formation by Regulating NRF2-Mediated Autophagy Dysfunction and Ferroptosis

**Figure 7:**
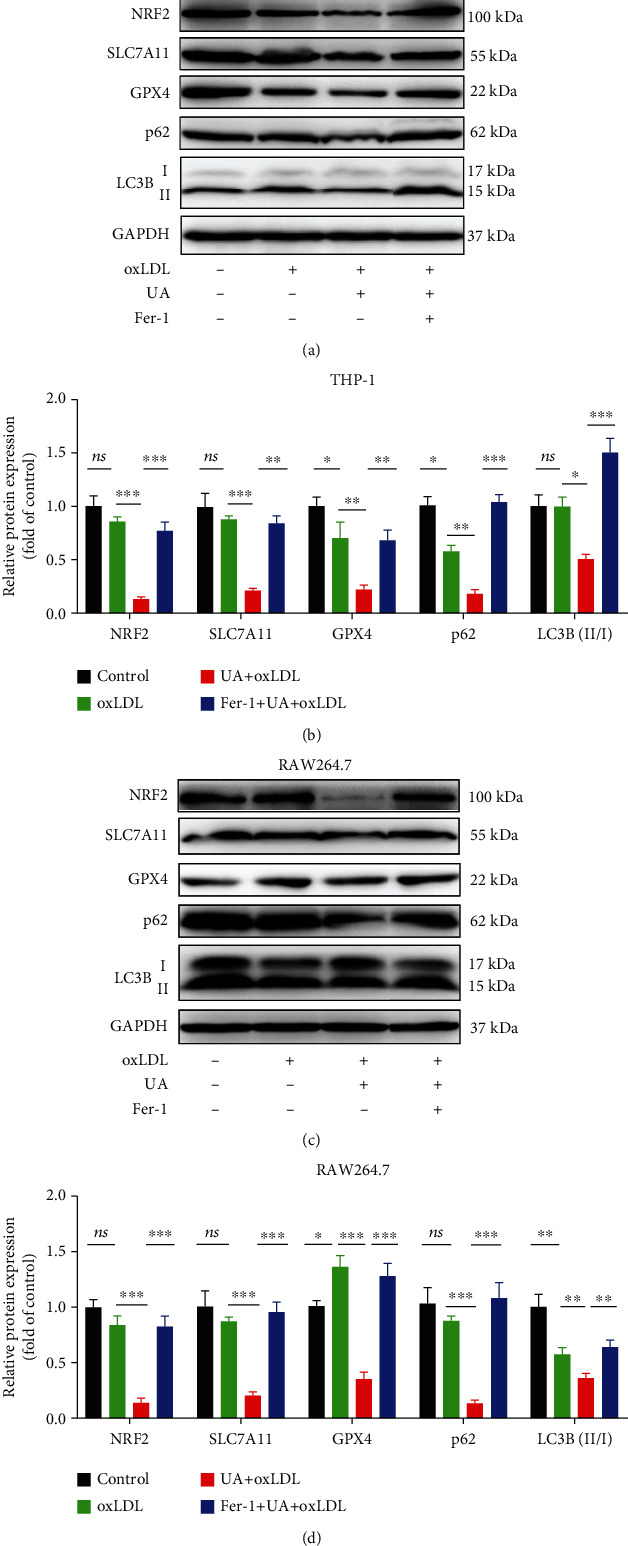
A ferroptosis inhibitor (Fer-1) reverses HUA-induced foam cell formation by targeting NRF2-mediated autophagy dysfunction and ferroptosis. THP-1 and RAW264.7 cells were treated with oxLDL (100 *μ*g/ml) or coincubated with UA (15 mg/dl) with or without Fer-1 (2 *μ*M) for 24 h. Western blot assay was used to detect the protein level of the NRF2/SLC7A11/GPX4 signaling pathway and autophagy-related proteins (LC3B and p62). (a and c) Representative western blot images of NRF2, SLC7A11, GPX4, LC3B, and p62. (b and d) Quantification of NRF2, SLC7A11, GPX4, LC3B, and p62 protein levels. Data are means ± SD, *n* = 3. Fer-1: ferrostatin-1; HUA: high level of uric acid; oxLDL: oxidized low-density lipoprotein; UA: uric acid. ns indicates no significance. ^∗^*P* < 0.05, ^∗∗^*P* < 0.01, and ^∗∗∗^*P* < 0.001.

To confirm the role and molecular mechanism of ferroptosis involved in HUA-induced foam cell formation, we used the specific inhibitor of ferroptosis, Fer-1. As shown in Figures 3 and 4, Fer-1 treatment reversed HUA-induced foam cell formation and inhibited HUA-induced ferroptosis in foam cells. In addition, the protein level of the NRF2/SLC7A11/GPX4 signaling pathway was decreased by HUA and restored by Fer-1 treatment (Figures 7(a)–7(d)). Consistently, Fer-1 restored the protein level of the autophagy-related proteins LC3B and p62 (Figures 7(a)–7(d)). These results support that HUA promotes atherosclerosis by modulating NRF2-mediated autophagy dysfunction and ferroptosis.

### 3.8. NRF2 Inducer (TBHQ) and Autophagy Activator (RAPA) Could Reverse the Inhibitory Effect of HUA on Foam Cell Survival

**Figure 8:**
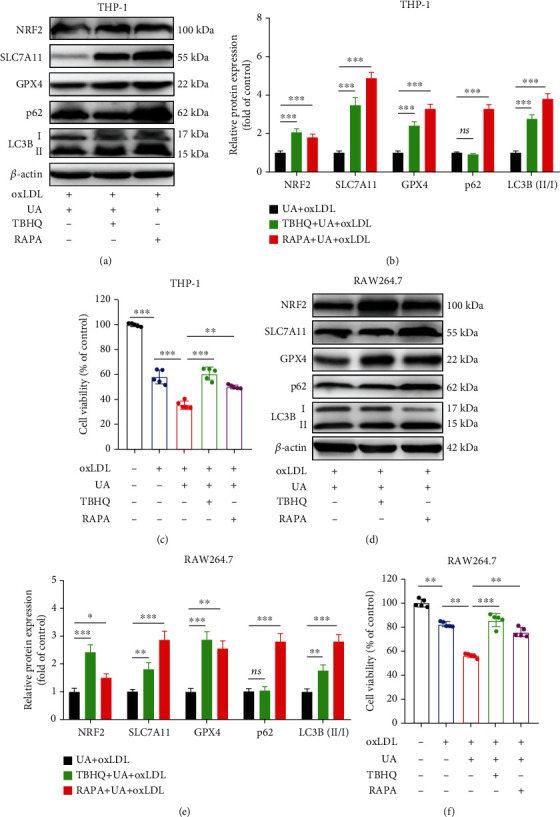
NRF2 inducer (TBHQ) and autophagy activator (RAPA) could reverse the inhibitory effect of HUA on foam cell survival. THP-1 and RAW264.7 cells were pretreated with TBHQ (10 *μ*M) or RAPA (10 *μ*M) for 0.5 h before coincubation with oxLDL (100 g/ml) and UA (15 mg/dl) for another 24 h. Western blot was used to detect the protein level of NRF2, LC3B, and p62. A CCK-8 kit was used to assay the cell viability of foam cells. (a and d) Representative western blot images of NRF2, SLC7A11, GPX4, LC3B, and p62. (b and e) Quantification of NRF2, SLC7A11, GPX4, LC3B, and p62 protein levels. (c and f) The cell viability of foam cells. Data are means ± SD, *n* = 3 − 5. HUA: high level of uric acid; oxLDL: oxidized low-density lipoprotein; RAPA: rapamycin; TBHQ, tertbutyl hydroquinone. ns indicates no significance. ^∗^*P* < 0.05, ^∗∗^*P* < 0.01, and ^∗∗∗^*P* < 0.001.

To further study the role of NRF2 or autophagy in HUA-induced ferroptosis, we used TBHQ and RAPA to activate NRF2 and autophagy in foam cells, respectively. TBHQ significantly enhanced the protein level of NRF2, along with SLC7A11 and GPX4 (Figures 8(a) and 8(b) and 8(d) and 8(e)) and reversed the inhibitory effect of HUA on foam cell survival (Figures 8(c) and 8(f)). Similarly, pretreatment with RAPA significantly activated autophagy, as evidenced by increased protein levels of LC3B and p62 (Figures 8(a) and 8(b) and 8(d) and 8(e)), and reversed the HUA-induced ferroptosis (Figures 8(c) and 8(f)). In sum, all these results further support that HUA promotes atherosclerosis by targeting NRF2-mediated autophagy dysfunction and ferroptosis.

## 4. Discussion

**Figure 9:**
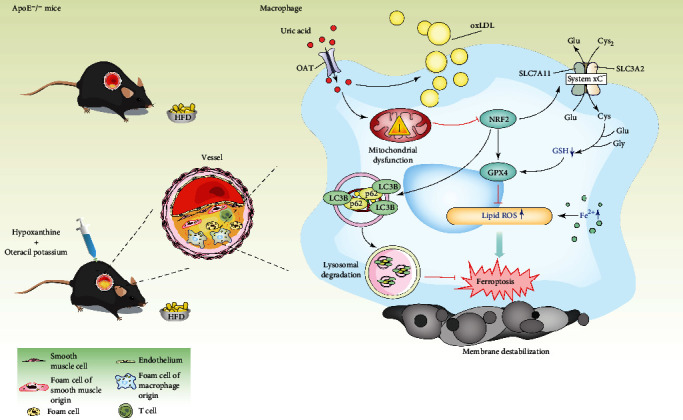
Schematic illustration of HUA promoting atherosclerosis by triggering ferroptosis in macrophage-derived foam cells. HUA induces mitochondrial dysfunction and inhibits NRF2 signaling, which may in turn impair macrophage autophagy and increase foam cell ferroptosis, thereby accelerating atherosclerosis. GPX4: glutathione peroxidase; GSH: glutathione; HFD: high fat diet; HUA: high level of uric acid; NRF2: nuclear factor erythroid 2-related factor 2; OAT: organic anion transporter; oxLDL: oxidized low-density lipoprotein; ROS: reactive oxygen species; UA: uric acid.

Collectively, in this study, we found that HUA impaired mitochondrial function, decreased cell viability and GSH level, and increased lipid ROS levels and iron accumulation in macrophage-derived foam cells, which were all reversed by Fer-1 treatment. Also, HUA inhibited the autophagy of foam cells and the protein level of the NRF2/SLC7A11/GPX4 signaling pathway, which were also reversed by Fer-1. HUA promoted the formation of atherosclerotic plaque and foam cells in ApoE^−/−^ mice and macrophages, respectively. In addition, NRF2-mediated autophagy dysfunction and ferroptosis may be involved in the occurrence and progression of hyperuricemia-associated ASVD (Figure 9).

ASVD has become one of the leading causes of death worldwide [47, 48]. An increasing number of epidemiological and clinical studies have shown hyperuricemia strongly associated with the occurrence and progression of many metabolic diseases, including atherosclerosis, hypertension, diabetes, and chronic kidney disease [3, 49–54]. The prevalence of hyperuricemia is increasing and in younger people worldwide. About 177 million people have hyperuricemia in China, nearly 60% of whom are aged 18 to 35 years [55]. Thus, hyperuricemia has become the fourth largest risk factor for atherosclerosis after hypertension, diabetes mellitus, and hyperlipidemia. ASVD associated with hyperuricemia increases the global healthcare burden.

Elevated sUA level may impair endothelial dysfunction by inflammation and oxidative stress, thus forming unstable lipid plaques in arteries and eventually leading to atherosclerosis [53, 56]. However, the molecular mechanisms by which HUA promotes atherosclerosis are far from clear. Therefore, the current treatment of hyperuricemia-associated ASVD is limited, and there is an urgent need to discover new mechanisms and provide new therapeutic targets.

Ferroptosis is a newly discovered type of regulated cell death mainly mediated by iron-dependent lipid peroxidation and has contributed to many pathological processes including ASVD, cancer development, neurodegenerative disease, and acute kidney injury [11, 26, 38, 39]. Accumulating evidence suggests that HUA can promote oxidative activity and increase the production of ROS [37, 57]. Therefore, we speculated that HUA may promote the progression of atherosclerosis by regulating ferroptosis in foam cells. In this study, we found that HUA promotes the formation of atherosclerotic plaque and foam cells in ApoE^−/−^ mice and macrophages, respectively. HUA decreased cell viability and GSH level and increased lipid ROS levels and iron accumulation in foam cells, which were all reversed by Fer-1 treatment. Immunofluorescence results and western blot analysis also confirmed that HUA inhibited the protein level of GPX4 and SLC7A11, which are critical key factors in the processing of ferroptosis in ApoE^−/−^ mice and macrophage-derived foam cells. Thus, HUA can promote atherosclerosis by regulating ferroptosis *in vivo* and *in vitro*.

Recent studies have shown that ferroptosis is an autophagic cell death process [34, 58, 59]. Autophagy plays a key role in regulating ferroptosis because of its ability to regulate cellular iron homeostasis and cellular ROS generation [35, 60]. Dysfunction of autophagy in macrophages contributes to atherosclerosis. Impaired autophagy-lysosomal degradation system leads to lipid accumulation, facilitating atherosclerotic plaque, while enhancing autophagy mitigates atherosclerosis development [61–66]. Consistently, we found that HUA suppressed the protein levels of LC3B and p62, which are markers of autophagic status, in macrophage-derived foam cells. That is, HUA impaired autophagy and increased the lipid accumulation in macrophages. Interestingly, Fer-1 treatment restored the HUA-inhibited protein levels of LC3B and p62. Additionally, an autophagy activator (RAPA) could reverse the inhibitory effect of HUA on foam cell survival. This result was consistent with a previous report showing that autophagy directly regulates SLC7A11, thereby maintaining tumor cell growth and proliferation [60]. In addition to SLC7A11, selective autophagy also regulates ferroptosis by degrading GPX4 [67, 68]. Therefore, autophagy-dependent ferroptosis plays an important role in tumorigenesis and development. The role of autophagy in ferroptosis was inconsistent with our current study, in which we showed that HUA inhibited macrophage autophagy and increased ferroptosis, thereby promoting the formation of foam cells. The role of selective autophagy (activation or inhibition) in ferroptosis might be tissue- or cell-specific. Although the role of autophagy dysfunction in ferroptosis needs further investigation, however, our data clearly indicate that HUA promotes foam cell formation by impairing macrophage autophagy and increasing ferroptosis.

NRF2 is considered a master antioxidant regulator, playing a crucial role in maintaining redox and metabolic homeostasis by regulating cellular antioxidants [19, 20]. In addition to the NRF2 signaling pathway, autophagy is another major intracellular defense system to combat oxidative damage and maintain homeostasis in mammals [69]. p62 is an ubiquitin-binding autophagy receptor protein that can connect to the NRF2 pathway and autophagy [32]. Increasing evidence has emerged for the important role of NRF2 in regulating ferroptosis [21]. Certain ferroptosis activators (erastin and sorafenib) can induce an interaction between p62 and KEAP1, thus activating NRF2 and the expression of antiferroptosis genes [70]. NRF2 transcription regulates several ferroptosis-related genes, including iron metabolism genes (HMOX1, FTH1, and FPN) [42, 71–74], a GSH synthesis- and release-related gene (SLC7A11) [22, 74, 75], and antioxidant genes (GPX4, NQO1 (NAD (P) H quinone dehydrogenase 1), TXNRD1, etc.) that enhance resistance to ferroptosis [70, 76, 77]. Thus, NRF2 plays a central role in the transcriptional regulation of ferroptosis. We found that HUA impaired mitochondrial function and inhibited the protein level of NRF2 in foam cells, as well as downstream SLC7A11 and GPX4. Fer-1 could alleviate the mitochondrial damage and reverse the HUA-reduced protein level of the NRF2/SLC7A11/GPX4 signaling pathway. Furthermore, an NRF2 inducer (TBHQ) could reverse the inhibitory effect of HUA on foam cell survival. This result was consistent with a previous study [33] showing that NRF2 activation by TBHQ suppressed diabetes-driven atherosclerosis *in vivo*. Thus, HUA may promote atherosclerosis by modulating NRF2-mediated autophagy dysfunction and ferroptosis.

## 5. Conclusion

In conclusion, our results show that HUA inhibits NRF2 and increases ferroptosis, thereby promoting the formation of atherosclerotic plaques in ApoE^−/−^ mice. In addition, HUA impaired mitochondrial function and autophagy, decreased cell viability and GSH level, and increased lipid ROS levels and iron accumulation in macrophage-derived foam cells *in vitro*. Furthermore, Fer-1 could reverse the protein level of the NRF2/SLC7A11/GPX4 signaling pathway and autophagy inhibited by HUA, thus decreasing ferroptosis and inhibiting the formation of foam cells. These findings indicate that HUA promotes atherosclerosis by targeting NRF2-mediated autophagy dysfunction and ferroptosis. Additionally, the inhibition of ferroptosis or activation of NRF2 could alleviate the progression of hyperuricemia-associated ASVD. Certain ferroptosis inhibitors or NRF2 activators may be potential targets for AVSD treatment. Nevertheless, further study is needed to confirm whether UA-lowering treatment can inhibit the progression of atherosclerosis by regulating ferroptosis.

## Figures and Tables

**Table 1:** qPCR primer sequences.

Gene	Species	Sense/antisense	Sequence (5′-3′)
*CD36*	Human	Sense	TTGATGTGCAAAATCCACAGG
		Antisense	TGTGTTGTCCTCAGCGTCCT
*ABCA1*	Human	Sense	ACCCACCCTATGAACAACATGA
		Antisense	GAGTCGGGTAACGGAAACAGG
*ABCG1*	Human	Sense	ATTCAGGGACCTTTCCTATTCGG
		Antisense	CTCACCACTATTGAACTTCCCG
*NFE2L2*	Human	Sense	TCAGCGACGGAAAGAGTATGA
		Antisense	CCACTGGTTTCTGACTGGATGT
*SLC7A11*	Human	Sense	TCTCCAAAGGAGGTTACCTGC
		Antisense	AGACTCCCCTCAGTAAAGTGAC
*GPX4*	Human	Sense	GAGGCAAGACCGAAGTAAACTAC
		Antisense	CCGAACTGGTTACACGGGAA
*CD36*	Mouse	Sense	ATGGGCTGTGATCGGAACTG
		Antisense	GTCTTCCCAATAAGCATGTCTCC
*ABCA1*	Mouse	Sense	GCTTGTTGGCCTCAGTTAAGG
		Antisense	GTAGCTCAGGCGTACAGAGAT
*ABCG1*	Mouse	Sense	CTTTCCTACTCTGTACCCGAGG
		Antisense	CGGGGCATTCCATTGATAAGG
*NFE2L2*	Mouse	Sense	TCTTGGAGTAAGTCGAGAAGTGT
		Antisense	GTTGAAACTGAGCGAAAAAGGC
*SLC7A11*	Mouse	Sense	GGCACCGTCATCGGATCAG
		Antisense	CTCCACAGGCAGACCAGAAAA
*GPX4*	Mouse	Sense	GCCTGGATAAGTACAGGGGTT
		Antisense	CATGCAGATCGACTAGCTGAG
*β-Actin*	Human	Sense	AGCGAGCATCCCCCAAAGTT
		Antisense	GGGCACGAAGGCTCATCATT
*β-Actin*	Mouse	Sense	GCAGGAGTACGATGAGTCCG
		Antisense	GGGTGTAAAACGCAGCTCAG

**Table 2:** Body weight and serum biochemical profiles of ApoE^−/−^ mice.

Parameters	SLD (*n* = 6)	HFD (*n* = 6)	HUA+HFD (*n* = 6)
Body weight (g)	27.22 ± 1.03	29.17 ± 1.52^∗^	27.0 ± 0.95^+^
sUA (*μ*mol/l)	247.3 ± 35.43	490.20 ± 106.00^∗∗^	1066.00 ± 180.90^+++^
TG (mmol/l)	1.20 ± 0.51	3.22 ± 0.19^∗∗∗^	3.39 ± 0.52
TC (mmol/l)	5.79 ± 1.41	43.09 ± 1.08^∗∗∗^	43.21 ± 2.21
HDL-C (mmol/l)	3.36 ± 0.42	2.74 ± 0.82	5.05 ± 1.13^+^
LDL-C (mmol/l)	1.11 ± 0.41	18.13 ± 1.67^∗∗∗^	13.94 ± 1.64^+^

Data are means ± SD. HDL-C: high-density lipoprotein-cholesterol; HFD: high fat diet; HUA+HFD: high level of uric acid and high fat diet; LDL-C: low-density lipoprotein-cholesterol; SLD: standard laboratory diet; sUA: serum uric acid; TC: total cholesterol; TG: triglycerides. ^∗^*P* < 0.05, ^∗∗^*P* < 0.01, and ^∗∗∗^*P* < 0.001 compared to SLD; ^+^*P* < 0.05 and ^+++^*P* <0.001 compared to HFD.

## Data Availability

The data used to support the findings of this study are available from the corresponding author upon request.

## Abbreviations

ABCA1: ATP-binding cassette transporter A1
ABCG1: ATP-binding cassette transporter G1
AFI: Average fluorescence intensity
ASVD: Atherosclerotic vascular disease
CCK-8: Cell counting kit-8
DAPI: 4′,6-diamidino-2-phenylindole
FCM: Flow cytometry
Fer-1: Ferrostatin-1
GPX4: Glutathione peroxidase
GSH: Glutathione
HDL-C: High-density lipoprotein-cholesterol
HFD: High fat diet
HUA: High level of uric acid
HUA + HFD: High level of uric acid and high fat diet
LDL-C: Low-density lipoprotein-cholesterol
MDA: Malondialdehyde
MMP: Mitochondrial membrane potential
NFE2L2/NRF2: Nuclear factor erythroid 2-related factor 2
oxLDL: Oxidized low-density lipoprotein
PMA: Phorbol 12-myristate 13-acetate
PVDF: Polyvinylidene difluoride
qPCR: Quantitative PCR
RAPA: Rapamycin
ROS: Reactive oxygen species
SLD: Standard laboratory diet
sUA: Serum uric acid
TBHQ: Tertbutyl hydroquinone
TC: Total cholesterol
TEM: Transmission electron microscopy
TG: Triglycerides.
